# Physicochemical Characteristics of Porous Starch Obtained by Combined Physical and Enzymatic Methods, Part 1: Structure, Adsorption, and Functional Properties

**DOI:** 10.3390/ijms25031662

**Published:** 2024-01-29

**Authors:** Monika Sujka, Agnieszka Ewa Wiącek

**Affiliations:** 1 Department of Analysis and Food Quality Assessment, Faculty of Food Sciences and Biotechnology, University of Life Sciences in Lublin, Skromna 8, 20-704 Lublin, Poland; 2 Department of Interfacial Phenomena, Faculty of Chemistry, Maria Curie-Skłodowska University, Maria Curie-Skłodowska Sq.3, 20-031 Lublin, Poland

**Keywords:** porous starch, specific surface area, adsorption, ultrasound, enzymatic treatment, surface charge, zeta potential

## Abstract

Porous starch can be applied as an adsorbent and encapsulant for bioactive substances in the food and pharmaceutical industries. By using appropriate modification methods (chemical, physical, enzymatic, or mixed), it is possible to create pores on the surface of the starch granules without disturbing their integrity. This paper aimed to analyze the possibility of obtaining a porous structure for native corn, potato, and pea starches using a combination of ultrasound, enzymatic digestion, and freeze-drying methods. The starch suspensions (30%, *w*/*w*) were treated with ultrasound (20 kHz, 30 min, 20 °C), then dried and hydrolyzed with amyloglucosidase (1000 U/g starch, 50 °C, 24 h, 2% starch suspension). After enzyme digestion, the granules were freeze-dried for 72 h. The structure of the native and modified starches were examined using VIS spectroscopy, SEM, ATR-FTIR, and LTNA (low-temperature nitrogen adsorption). Based on the electrophoretic mobility measurements of the starch granules using a laser Doppler velocimeter, zeta potentials were calculated to determine the surface charge level. Additionally, the selected properties such as the water and oil holding capacities, least gelling concentration (LGC), and paste clarity were determined. The results showed that the corn starch was the most susceptible to the combined modification methods and was therefore best suited for the production of porous starch.

## 1. Introduction

The growing demand for natural, cheap, non-toxic, and biodegradable adsorbents for the food industry has resulted in increased interest among researchers in starch as a raw material for their production. Native starch naturally occurs in the form of semi-crystalline granules of round, spherical, polygonal, elongated, and kidney shapes, depending on the botanical source of starch. Porous starch may be obtained by enzymatic (hydrolysis with e.g., α-amylase (EC 3.2.1.1), amyloglucosidase (EC 3.2.1.3), glycogen branching enzyme (EC 2.4.1.18)), chemical (e.g., solvent exchange, crosslinking, acid hydrolysis) or physical (e.g., extrusion, ultrasonic treatment, pulsed electric field) methods, or a combination of them [1,2,3,4,5,6,7,8,9,10].

Parameters characterizing the texture of food materials and influencing the mechanical, diffusion and sensory properties of food include, among others, porosity, pore size and pore size distribution [11]. According to the IUPAC classification, there are three groups of pores in solid materials: (a) macropores (width > 50 nm), (b) mesopores (2 nm < width < 50 nm), (c) micropores (width < 2 nm) [12]. Pores are a natural feature of starch granules, although they can also be formed during mechanical or enzymatic processing of starch [9,13,14,15]. Low-temperature nitrogen adsorption belongs to the most frequently used methods for determining the starch porosity [2,5,13,14,15,16]. This method allows to determine the porosity of the material in the entire range of mesopores and the lower range of macropores (pore diameter 1.7–300 nm) and it involves determining the nitrogen adsorption isotherm at the temperature of liquid nitrogen and calculating the capacity of the monolayer based on the BET adsorption isotherm [13,14,17,18]. Porosity of starch granules influences their functional properties and reactivity, because the presence of pores, channels and cavities increases the surface area that is potentially available for molecules of chemical compounds and amylolytic enzymes (i.e., the greater the specific surface area of the granules, the greater the susceptibility to amylolysis) [15,16,19,20]. Porous starch has many applications in the food industry, mainly as a carrier for ingredients such as polyphenols [21,22], essential oils [23], flavors [24], or probiotics [25].

Starch also has very important biological functions in the human body. It is a source of energy for the central nervous system and red blood cells, it is also necessary in the synthesis of amino acids and the oxidation of fatty acids in the body. Starch contains hydroxyl groups and simple glycosidic bonds that can be easily modified to improve its functional properties or to incorporate specific new features enhancing cooking properties, gel clarity, texture, adhesion, and film formation, increasing freeze–thaw stability or reducing syneresis, retrogradation, and gelling tendencies. Starch amphiphilicity, hydrophobicity, mechanical strength, and thermal stability are among the properties that can be improved as a result of starch modification. Regarding such many aspects wettability tests using probe liquids of various chemical nature may be helpful. Based on the wettability parameters, conclusions can be drawn regarding the potential interactions of starch with biological membranes, physiological fluids, and other substances [26,27].

The purpose of this study was to use three methods of modification: ultrasound, enzymatic hydrolysis, and freeze-drying to prepare porous starch from starches of different botanical origin. Their structural, adsorption, and functional properties, were analysed to select the most effective porous matrix for adsorption of valuable bioactive substances. In assessing the degree of starch modification, changes in the surface charge of granules before and after modification with combined methods, the electrokinetic zeta potential, which indirectly may also inform about changes in the stability of the system and preliminary wettability test, were used.

## 2. Results and Discussion

### 2.1. Structure and Morphology of Native and Modified Starch

The first step in assessing the impact of chemical, physical or enzymatic modifications on the structure of starch may be the analysis of its complex with iodine. For example, blue value (BV) and λ_max_ provide information on the degree of polymerization of amylose and the average chain length (CL) of amylose and amylopectin [28]. Results of the following determinations: degree of hydrolysis, BV, A_680_/A_545_ ratio, and λ_max_ are presented in Table 1. Among the starches tested, the most susceptible to hydrolysis by amyloglucosidase was corn starch, followed by pea starch, and the most resistant was potato starch. These results are consistent with literature data [29]. Iodine forms a coloured complex with linear chains which gradually shifts from red to blue based on the length of the available chains. The value of λ_max_ for long chains (amylose-like chains) is >610 nm, whereas for short chains (amylopectin-like chains) it is 530–575 nm [30]. Pea starch, unlike corn and potato starches, had the A_680_/A_545_ ratio over 1 which means that it contained a higher portion of amylose-like chains than amylopectin-like chains. This is correlated also with the highest λ_max_ and BV for this starch. For corn and potato starches, the content of both types of chains was almost equal.

As seen in Table 1, the values of all parameters related to the starch-iodine complex decreased after the treatment of granules with three modification methods: ultrasound, enzymatic hydrolysis, and freeze-drying. This means that as a result of physical forces and the enzyme, partial depolymerization of starch occurred [31,32].

**Figure 1:**
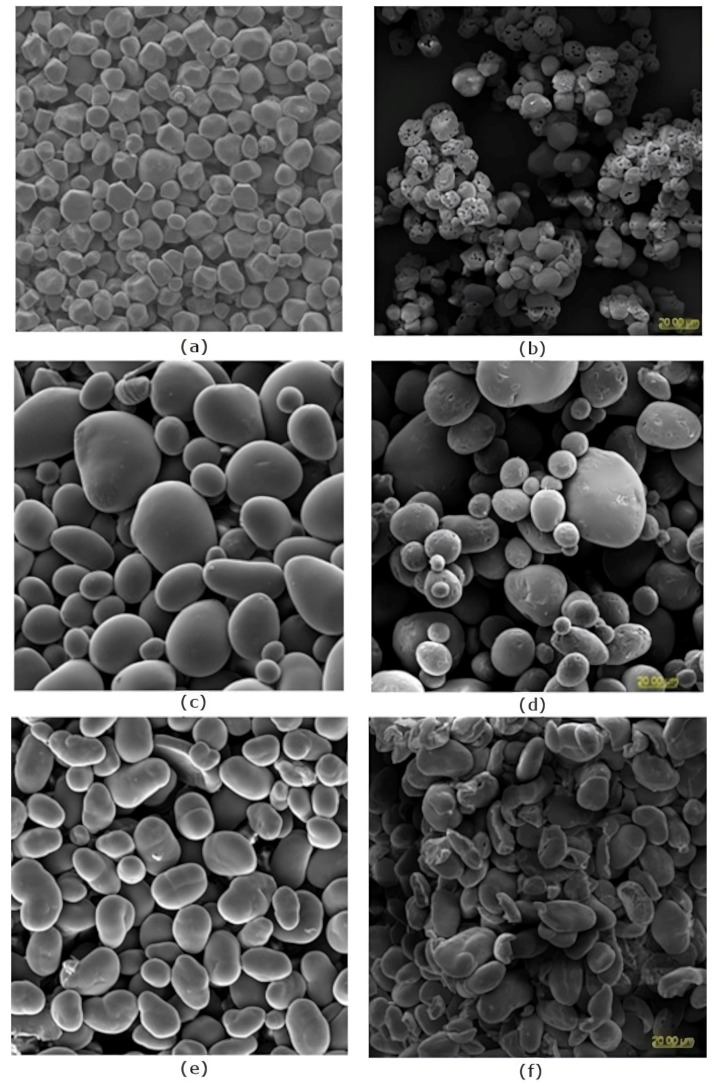
SEM micrographs of native (**a**,**c**,**e**) and modified (**b**,**d**,**f**) starch granules: (**a**,**b**)-corn starch, (**c**,**d**)-potato starch, (**e**,**f**)-pea starch (magnification 1200×).

Figure 1 shows SEM micrographs of starch granules before and after the three-step modification. Native starch granules have a shape typical of botanical origin and are rather smooth, except for corn starch, which has a rough surface (Figure 1a,c,d). In the case of modified starches, holes on the surface, giving the granules a spongy structure, are present in corn starch (Figure 1b). Distinct depressions are also visible on the surface of potato starch (Figure 1d), while the pea starch granules are mainly cracked and fragmented (Figure 1e). Surface roughness, pores, and cracks were induced by the force of ultrasonic cavitation, enzymatic digestion and local explosive release of water vapour from the accumulated pressure inside the rigid granules under vacuum conditions during freeze-drying [14,29,33]. All these irregularities increase the surface area potentially available for adsorption.

**Figure 2:**
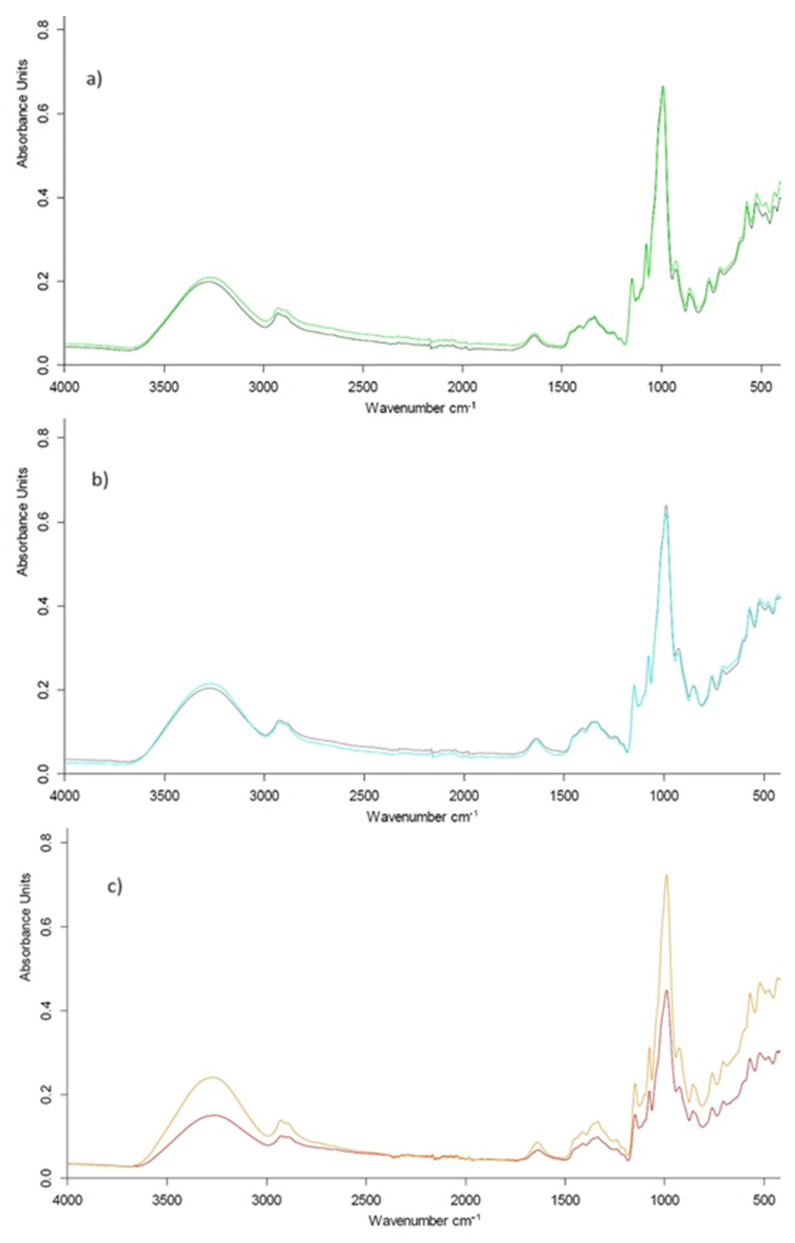
ATR-FTIR spectra of starches: (**a**) native corn (light green) and modified corn (dark green), (**b**) native potato (light blue) and modified potato (dark blue), (**c**) native pea (orange) and modified pea (red).

Figure 2 represents ATR-FTIR spectra of starches obtained in the range of 400–4000 cm^−1^. The absorption peaks for native starches were similar to those of modified starches indicating that no new chemical bonds were formed during the modification. In the FTIR spectrum, the absorption band of starch at 1047 cm^−1^ is associated with the structural features of the crystalline region, representing the short-range ordered structure of the starch molecule, whereas the absorption band at 1022 cm^−1^ is associated with the structural features of the non-crystalline region of starch. The next absorption band at 995 cm^−1^ reflects the double helix structure in the crystalline regions [34].

The absorbance ratios of 1047 cm^−1^/1022 cm^−1^ and 1022 cm^−1^/995 cm^−1^ obtained for native and modified are gathered in Table 2. The results revealed that the ratio of 1047/1022 cm^−1^ was slightly higher and the ratio of 1022/995 cm^−1^ was slightly lower for modified starches than for their native counterparts.

This means that the amorphous area of starch was reduced as a consequence of the use of combined modification methods. This is in line with the observations of Lacerda et al. [35].

### 2.2. Specific Surface Area and Pore Characteristics of Native and Modified Starch

Table 3 shows the results of the determination of specific surface area (S_BET_), volume and average diameter of pores present in the starch granules. The values of S_BET_ for native starches are in the range of 0.02–0.56 m^2^/g and for modified starch between 0.8 and 4.24 m^2^/g. These values are consistent with those obtained in previous studies [13,14,36]. The highest S_BET_ among native starches and the rough surface observed in SEM micrographs for corn starch (Figure 1b) correlate well with its greatest susceptibility to hydrolysis with amyloglucosidase. Natural pores on the surface of native starch granules (such as corn starch) can facilitate the formation of pores by enzymes [37]. Using combined methods of modification led to an increase in the volume of mesopores for all examined starches and the average pore diameter of mesopores for corn starch. For pea and potato starches the value of the latter parameter is lower, especially in the case of potato starch, which means that as a result of the modification, the number of pores with smaller diameters increased.

### 2.3. Zeta Potential Calculation of the Starch Suspensions and Preliminary Wettability Test

The electrophoretic mobility of suspensions of various types of native and modified starch was measured using the laser Doppler velocimeter method, and zeta potentials were calculated on this basis to determine the level of surface charge. As shown in Table 4 both native and modified potato starch and pea starch provided negative surface charges, which were visualized by zeta potential values.

These values are relatively small and mean values oscillate within the range from −1.6 to 2.7 mV. The change after modification is noticeable, but within the limits of statistical error. However, the values obtained for the dispersion from the third type of starch (corn starch) are completely different. Native corn starch is positively charged, and its surface had a zeta potential of about 10.5 mV. For modified starch, the absolute value of zeta potential increased when compared with native corn starch. Average values of 17.7 mV were obtained. Only in this one case such a large change was achieved after modification. This leads to conclusion that corn starch can be modified relatively easily and as was early mentioned it correlates well with its susceptibility to hydrolysis with amyloglucosidase.

It is well known that starch granules are held together by hydrogen bonds and interchain hydrophobic bonds. During hydrolysis possible process may be the breaking of hydrogen bonds between poly-(1 → 4)-glucan chains in crystallites and subsequent the hydrophobic bonds. After breaking hydrogen bonds, the polar groups probably hide inside and the hydrophobic parts remain outside starch granules, so a decrease in zeta potential is noticeable (Table 4). In every starch glucose unit there are three–OH groups, however, not all of them can be present on the granule surface. The hydroxyl groups are the only polar groups available in the biopolymer chain, which are capable of interactions. During hydrolysis the most hydrophobic parts of the starch chain are directed into air. However, steric limitations cause that some polar groups are also present on the surface. The starch granule surface tends to reach maximal hydrophobic character with polar domains created by the functional glucose groups. It is most probable that the branched chain of amylopectin is directed into air and calculated zeta potential changes after modification is probably caused by the increased amount of –OH groups on the starch surface. Taking into account only the zeta potential results, it can be concluded that the modification was most effective in the case of corn starch. This correlates with the porosity changes summarized in Table 3. The specific surface area of corn starch increased threefold and the volume of mesopores increased almost fourfold. In the case of potato and pea starches, these changes were not so noticeable.

The next idea of our research will be, in addition to increasing porosity, adding antioxidant properties through the use of gallic acid. Research that is already promising at this stage will be discussed in Part 2 of the manuscript. Improvement of antioxidant activity of starch film by phenolic acid was described early by Hao et al. [38]. It is important that phenolic acids exert also antimicrobial activity, which is considered beneficial to human health. Lately authors stated that in the treatment with a higher amount of oxidant, most polysaccharides in the starch were degraded to oligosaccharides. Similar process is possible during hydrolysis. It would be reasonable to expect that oxidized starch with a higher oxidation would have a greater negative charge, while a higher amount of oxidant as described in recent papers [38,39,40,41,42] resulted in the higher solubility of starch. To check these properties for our three types of starch, the wettability measurements were performed for their dispersions deposited on glass plates analogous as in our previous papers [26,27,43]. These studies should be considered as preliminary. Milli-Q water was used during wettability measurements as a typical polar tested liquid. Water wettability of starch films before and after modification is also important due to another aspect, e.g., process of microorganism growth. After three-step modification, water wetting times for plates covered with modified starch suspensions increased noticeably, sometimes even several times. This is probably the result of the presence of additional polar groups on starch film surface. For a complete picture, similar tests should be carried out with test liquids of a different chemical nature, i.e., polar formamide and non-polar diiodomethane. These studies are being carried out using Washburn procedure, however due to their time-consuming nature will be described in detail in Part 2 of the manuscript. On the other hand, modified starch-based hydrogel can be also used as an adsorbent for food and non-food application. Therefore, detailed porosity tests were performed on all tested starches (discussed in the Section 2.2).

### 2.4. Functional Properties of Native and Modified Starches

Modification of starches by combined methods affected their functional properties. Results of WA, OA, LGC and paste clarity determinations are summarized in Table 5. Among native starches, corn starch has the highest water and oil absorption capacity, and LGC, while potato starch is characterized by the highest paste clarity. Modification generally caused an increase in the water absorption (1.3–1.5-fold) and oil absorption (1.1–1.4-fold), and a decrease in the paste clarity (approximately 3-fold) for all the examined starches. LGC of modified corn and potato starches are higher than before modification or stayed unchanged in the case of pea starch.

The increase in water and oil absorption results from the disruption of the hydrogen bonds between the amorphous and crystalline area. Moreover, this increase could also be caused by changes in the structure of the starch granule surface. It became more porous, less “packed”, making it easier to absorb water and oil [44]. So, despite the hydrophilic nature of starch, modified starches showed good capacity for oil absorption. Modification of corn and potato starch weakened the gelling properties of amylose to some extent, which is reflected in higher LGC values. In the case of paste clarity, scientists more often report that the value of this parameter increases after the action of e.g., acid hydrolysis or ultrasonics, as a result of partial depolymerization of starch [32,45]. However, there are also contrary reports e.g., lower paste clarity was found by Li et al. after ultrasonic treatment of pea starch [46]. The authors explained this by long-term processing, which produced more short amylose chains that were easier to accumulate, resulting in starch condensation. This study additionally used two other modification methods that could enhance this effect.

## 3. Materials and Methods

### 3.1. Materials

Starches (corn, potato, and pea) were purchased from Sigma-Aldrich Inc. (St. Louis, MO, USA), WPPZ S.A. (Luboń, Poland), and Roquette (Lestrem, France). Amyloglucosidase was obtained from Merck (Darmstadt, Germany). Distilled water from the Milli-Q system (resistivity ~18.2 MΩ × cm) for electrophoretic mobility and wettability measurements was used.

### 3.2. Methods

#### Modification of Starch with Ultrasounds, Enzymatic Hydrolysis, and Freeze-Drying

A 30% starch suspension in distilled water was prepared in a 100 mL beaker. The sample was exposed to ultrasound with a frequency of 20 kHz and a power of 170 W for 15 min at a temperature of 20 °C. During sonication, the sample was cooled by immersing the beaker in ice water. Then it was centrifuged (5000 rpm for 5 min), and the sediment was washed with 96% ethanol and left to dry. After drying, starch samples were used for enzymatic hydrolysis.

A portion of 15 mL of amyloglucosidase solution with an activity of 1000 U/g starch was added to a 2% starch suspension in citrate buffer at pH 4.2. The total sample volume was 300 mL. Hydrolysis was carried out at 50 °C for 24 h in a shaking incubator (150 rpm). After this time, the suspension was centrifuged (5000 rpm for 5 min), the supernatant was collected, and the sediment was washed with 96% ethanol. Then the sample was freeze-dried for 3 days at −45 °C in a vacuum (Labconco, Kansas City, MO, USA). The concentration of released glucose in the supernatant was determined by the enzymatic method (GOPOD) using the Megazyme kit (Bray, Ireland). The assay was performed in duplicate. Based on the results obtained, the degree of hydrolysis (%) was calculated.

### 3.3. Characteristics of Native and Modified Starch

#### 3.3.1. Analysis of Starch-Iodine Complexes

Blue value (BV) of the starch-iodine complex was determined based on the absorbance measured at 680 nm and calculated according to the procedure of Gilbert and Spragg [47]. Iodine absorption spectra were measured in the range of λ from 400 to 800 nm with a spectrophotometer V-630 (Jasco Analytical Instruments, Tokyo, Japan). Additionally, A_680_/A_545_ relations were calculated. Samples were analyzed in triplicate.

#### 3.3.2. Scanning Electron Microscopy (SEM)

Dried starch samples were applied to double-sided adhesive tape attached to an aluminum plate. They were then coated with a thin layer of gold using an Emitech K550X sputter coater (Quorum Technologies Ltd., Laughton, UK) and observed in a Vega2 microscope (Tescan, Brno, The Czech Republic) at an accelerating voltage of 10 kV. Images were recorded at 1200× magnification.

#### 3.3.3. ATR-FTIR Analysis

Mid-IR absorption spectra were obtained using attenuated total reflectance-Fourier transform infrared spectroscopy (ATR-FTIR) (Alpha II, Bruker Optics Inc., Billerica, MA, USA). Measurements were performed in damped total reflection mode using a Platinum ATR diamond crystal accessory. A pinch of the dried sample was placed directly on the crystal (with a contact surface diameter of 1.8 mm) and pressed against its surface. Spectra were collected by performing 36 spectral scans at a resolution of 4 cm^−1^ in the wavenumber range between 4000 and 400 cm^−1^. All spectral analyses were performed using OPUS 8.5 SP1 software (Bruker Optics Inc., Billerica, MA, USA).

#### 3.3.4. Low-Temperature Nitrogen Adsorption

Characteristics of pores of equivalent radius in the range of 1.7–300 nm was investigated based on nitrogen adsorption isotherms. The isotherms were measured using an apparatus ASAP 2420 (Micromeritics Inc., Norcross, GA, USA) at the temperature of liquid nitrogen (−195.85 °C) in the range of relative pressures p/p_0_ from 0.004 to 0.997. The measurements were performed on samples of 1 to 3 g, depending on the specific surface area. Before the measurements, starch samples were dried for 24 h in a vacuum at 100 °C, automatically desorbed, and flushed with pure helium. The volume of the pores was calculated with the use of computer software attached to the instrument.

The surface area of the investigated starches was evaluated from sorption isotherms of nitrogen using the BET method [17]. In the first step, the monolayer value (*a_m_*) was calculated within the range of p/p_0_ = 0.06–0.20 (from five measurement points) using a linear polynomial form of the BET equation. Equation (1) describes the relationship between relative pressure and the amount of adsorbed/desorbed liquid nitrogen. The slope of this relationship and the intercept were determined.

*a_m_* = 1/(*tgα* + *a*) (1)

where: *a*—adsorption, *a_m_*—monolayer value [cm^3^ N_2_/g dry mass].

The specific surface area was calculated from Equation (2):

*S_BET_* = (*a_m_* × *ω* × *L*)/*M* (2)

where: *a_m_* is a monolayer value (cm^3^/g dry mass), *L* is the Avogadro number (6.02 *×* 10^23^ molecules per mole), *M*—the molecular weight of nitrogen (28,013 g/mol), and *ω* is the molecular cross-section area (16.2 *×* 10^−20^ m^2^ for nitrogen molecule).

Pore characteristic was determined according to BJH method [48]. Samples were analyzed in triplicate. 

#### 3.3.5. Determination of the Zeta Potential

To perform electrophoretic mobility measurements, it was necessary to prepare a starch and modified starch dispersion of appropriate concentrations. Starch dispersions were measured for their electrophoretic mobility by laser Doppler velocimetry using a zeta potential analyzer (ZetaPlus-Bimass, Brookhaven Instrument Corporation, Holtsville, NY, USA). In each experiment, the same amount of different kinds of starch or starch after modification was dispersed in 100 mL of distilled water from the Milli-Q system (resistivity ~18.2 MΩ × cm) and then sonicated at 20 °C for 15 min (10,000 rpm, Silent Crush homogenizer, Heidolph). The samples were sonicated for 15 min to attain finely dispersed gel particles. The temperature of all samples was controlled at 20 °C. Initially, two starch weights, 0.3 g or 0.5 g, were tested, but ultimately, the higher weight was chosen because it turned out to be more suitable for further stages of the experiment, i.e., wettability measurements. The tests were carried out at time intervals of up to 2 h (15 min, 30 min, 60 min, 120 min) after the end of homogenization and after a week. The electrophoretic mobility of each sample was measured five times in each measurement.

#### 3.3.6. Preliminary Wettability Tests

Glass plates (primary glass, polished edges, optically smooth and clear, ca 76 × 26 × 1 mm, AS Polonia Ltd., Nottingham, UK) [49] were used as solid substrata for covering with native or modified starch dispersion at room temperature. About 4 mL of dispersion was slowly spread on the plate to obtain a homogeneous layer. Next, the plates were kept at room temperature for drying, and after that, were placed in desiccators filled with a silica gel until the measurements were performed, which was a maximum of a week. In the case of the powdered porous solids used in food or pharmaceutical industry, the degree of wetting should be described not by contact angle measurements, but rather by the kinetics of the wetting process, because it is difficult to achieve equilibrium. Therefore, often, the thin-layer wicking method is used regarding the penetration of a probe liquid into a thin, porous layer of the solid powder deposited, for example, on a metal, plastic, or glass support. Such a process may be described by Washburn’s equation, based on Poiseuille’s law [26]:

x^2^ = ΔG R t/2 η (3)

where: x^2^ is the square of penetration distance; t is the penetration time, η is the liquid viscosity, R is the effective radius of the intergranular capillaries that are formed in a porous layer or column of a powdered solid body, ΔG is the change in free energy (enthalpy) accompanying the replacement of the unit interface surface, i.e., the solid–gas and solid–liquid phases, during liquid movement (wetting) in a porous film.

Details of the methodology have been described in our previous papers [26,27,43].

The measurement of the time spreading of the probe liquids of different chemical characters provides important information about the starch wettability before and after modification of the relatively uncomplicated experimental procedure. Applying different kinds of probe liquids allows us to estimate liquid interfacial interactions. More advanced methods also allow us to determine the values of apparent surface free energy [26,27]. On the other hand, their changes reveal interesting information about the energetic properties of the investigated starch surface. The selection of liquids of different characters (strongly polar, less polar, or apolar) gives an overview of the various types of interactions, and enables a full description of the phenomenon of the wetting process. On this basis, the hydrophilic/hydrophobic nature of the tested surface for different kinds of starch can be determined, which consequently helps to select specific applications.

### 3.4. Functional Properties of Native and Modified Starch

#### 3.4.1. Oil Absorption (OA)

The starch sample (0.5 g) was weighed into a centrifuge tube and 10 mL of oil was added. The mixture was mixed for 30 s using a homogenizer at 10,000 rpm, and the mixer was rinsed with distilled water. The tubes were left to stand for 5 min and then mixed again. The tubes with their contents were centrifuged (3000 rpm) for 10 min. The unbound oil was gently poured off and the tubes were placed upside down on filter paper for 10 min, then weighed, and calculations were made.

OA = (a − d)/W × 100%(4)

where: OA—oil absorption (%), a—mass of tube with sample after centrifugation (g), d—mass of dry tube (g), W—sample mass (g).

The assay was performed in duplicate.

#### 3.4.2. Water Absorption (WA)

The starch sample (0.5 g) was weighed into a centrifuge tube and 12.5 mL of distilled water was added. The mixture was mixed for 30 s using a homogenizer at 10,000 rpm, and the mixer was rinsed with distilled water. The tubes with their contents were centrifuged (3000 rpm) for 10 min. The unbound water was gently poured off and the tubes were placed upside down on filter paper for 10 min, then weighed and calculations were made.

WA = (c − b)/W × 100%(5)

where: WA—water absorption (%), a—mass of tube with sample after centrifugation (g), d—mass of dry tube (g), W—sample mass (g).

#### 3.4.3. Clarity of Starch Pastes and the Minimum Concentration of Gel-Forming Starch Suspension (LGC)

A 1% starch suspension was prepared, then it was heated in a boiling water bath for 15 min, stirring occasionally, and cooled. The transmittance of the paste was measured at a wavelength of 650 nm against distilled water as a blank sample. The assay was performed in duplicate.

For LGC measurement, starch suspensions with concentrations of 2–10% (every 2%) were prepared in distilled water. The samples were heated in a boiling water bath for 15 min, stirring occasionally, then cooled and placed in a refrigerator (4 °C) for 2 h. After this time, it was assessed at which concentration the sample did not fall out of the inverted test tube. The assay was performed in duplicate.

### 3.5. Statistical Analysis

The obtained results of degree of hydrolysis, VIS spectroscopic determinations, and functional properties of the starch were subjected to statistical analysis in the astatsa.com online program. One-way analysis of variance (ANOVA) was used to compare the results, and then the Tukey post hoc test was used to determine the significance of differences between the group mean values. Statistical hypotheses were verified at the significance level of *p* < 0.05.

## 4. Conclusions

The modification of starch by three methods: ultrasound, hydrolysis with amyloglucosidase, and freeze-drying, improved the starch properties in terms of the porosity, oil and water absorption, paste clarity, surface charge, and wettability. The granules of the modified starches had a spongy structure (corn starch), were cracked and fragmented (pea starch), or showed only fissures and depressions on the surface (potato starch). The specific surface area and volume of the mesopores increased after modification for all the examined starches. The three-stage modification resulted in an increase in the average pore diameter only for the corn starch, while in the case of the potato and pea starch, more pores with a smaller average diameter were created. Changes in the electrokinetic potential and wettability parameters correlate quite well with changes in the porosity and are a good tool for assessing the processes occurring on the starch surface. The functional properties of the starches changed as a consequence of the modifications: the absorption of water and oil increased for all the examined starches, whereas the paste clarity decreased. In the case of the LGC, the modification caused an increase in the case of the corn and potato starch, while in the case of the pea starch, no effect was observed.

## Figures and Tables

**Table 1:** Degree of starch hydrolysis by amyloglucosidase and results of spectroscopic analysis of starch-iodine complexes obtained for native and modified starches.

Starch		Degree of Hydrolysis (%)	λ_max_	A_680_/A_545_	BV
Corn	native	-	592 ± 4 ^cd^	0.91 ± 0.06 ^ab^	0.79 ± 0.04 ^b^
	modified	48.28 ± 3.83 *^c^	576 ± 4 ^a^	0.80 ± 0.12 ^a^	0.65 ± 0.01 ^a^
Potato	native	-	586 ± 2 ^bc^	0.96 ± 0.16 ^ab^	0.85 ± 0.02 ^b^
	modified	5.41 ± 0.07 ^a^	581 ± 3 ^ab^	0.93 ± 0.22 ^ab^	0.82 ± 0.02 ^b^
Pea	native	-	608 ± 7 ^e^	1.17 ± 0.15 ^b^	1.07 ± 0.04 ^c^
	modified	24.00 ± 2.00 ^b^	603 ± 8 ^de^	1.02 ± 0.05 ^b^	1.05 ± 0.02 ^c^

* values are expressed as mean ± SD. Results with the same letter within a column are not significantly different (*p* < 0.05).

**Table 2:** IR ratio of the absorbances 1047/1022 (cm^−1^) and 1022/995 (cm^−1^) for native and modified starches.

Starch		IR Ratio 1047/1022 (cm^−1^)	IR Ratio 1022/995 (cm^−1^)
Corn	native	0.99 ± 0.01 *	1.01 ± 0.01
	modified	1.02 ± 0.02	0.98 ± 0.01
Potato	native	1.01 ± 0.02	0.99 ± 0.02
	modified	1.05 ± 0.01	0.95 ± 0.01
Pea	native	1.01 ± 0.02	0.99 ± 0.01
	modified	1.02 ± 0.02	0.97 ± 0.02

* values are expressed as mean ± SD.

**Table 3:** Specific surface area (S_BET_), volume, and average pore diameter for native and modified starches.

Starch		Specific Surface Area (m^2^/g)	Volume of Mesopores (cm^3^/g) × 10^−3^	Average Pore Diameter (nm)
Corn	native	0.56 ± 0.04 *	1.23 ± 0.06	8.72 ± 0.06
	modified	1.66 ± 0.08	4.24 ± 0.12	10.21 ± 0.04
Potato	native	0.02 ± 0.01	0.01 ± 0.01	33.87 ± 1.20
	modified	0.22 ± 0.02	0.80 ± 0.06	14.47 ± 0.19
Pea	native	0.16 ± 0.02	0.55 ± 0.04	14.01 ± 0.08
	modified	0.40 ± 0.06	1.24 ± 0.05	12.54 ± 0.10

* values are expressed as mean ± SD.

**Table 4:** Zeta potential for different kind of starches suspensions as a function of time.

Starch	Zeta Potential [mV]	15 min	30 min	60 min	120 min	Week	Mean
Corn	native	7.41 ± 3.2 *	10.58 ± 4.1	9.86 ± 2.6	7.66 ± 4.0	16.76 ± 3.8	10.45 ± 3.5
	modified	−13.55 ± 4.8	−8.85 ± 0.1	−20.14 ± 3.4	−28.06 ± 7.2	−17.8 ± 1.2	−17.7 ± 3.3
Potato	native	−3.60 ± 1.8	−3.36 ± 1.6	−1.96 ± 0.9	−1.56 ± 0.5	−1.76 ± 0.7	−2.45 ± 1.1
	modified	−1.16 ± 0.9	−1.58 ± 0.7	−1.11 ± 0.2	−2.41 ±0.5	−1.76 ± 0.4	−1.60 ± 0.5
Pea	native	−0.74 ± 0.3	−2.73 ± 1.0	−1.89 ± 0.8	−1.15 ± 0.2	−1.52 ± 0.1	−1.61 ± 0.5
	modified	−1.36 ± 0.5	−1.38 ± 1.8	−4.01 ± 1.5	−3.14 ± 2.1	−3.58 ± 1.8	−2.69 ± 1.5

* values are expressed as mean ± SD.

**Table 5:** Functional properties of native and modified starches.

Starch		WA (%)	OA (%)	LGC (%)	Paste Clarity (%)
Corn	native	194.65 ± 0.64 ^*c^	173.05 ± 3.46 ^c^	6 ^b^	21.50 ± 0.50 ^e^
	modified	261.15 ± 1.34 ^e^	237.55 ± 4.03 ^e^	8 ^c^	8.30 ± 0.42 ^b^
Potato	native	174.85 ± 3.47 ^a^	152.10 ± 1.70 ^a^	4 ^a^	74.85 ± 2.62 ^f^
	modified	229.75 ± 2.61 ^d^	168.95 ± 2.05 ^bc^	6 ^b^	25.25 ± 0.92 ^d^
Pea	native	186.20 ± 0.71 ^b^	163.15 ± 0.50 ^b^	4 ^a^	15.25 ± 0.21 ^c^
	modified	286.95 ± 0.64 ^f^	206.45 ± 6.01 ^d^	4 ^a^	5.20 ± 0.00 ^a^

* values are expressed as mean ± SD. Results with the same letter within a column are not significantly different (*p* < 0.05).

## Data Availability

The data presented in this study are available on request from the corresponding authors.
